# Are disseminated tumor cells in bone marrow and tumor-stroma ratio clinically applicable for patients undergoing surgical resection of primary colorectal cancer? The Leiden MRD study

**DOI:** 10.1007/s13402-016-0296-2

**Published:** 2016-09-09

**Authors:** F. J. Vogelaar, G. W. van Pelt, A. M. van Leeuwen, J. M. Willems, R. A. E. M. Tollenaar, G. J. Liefers, W. E. Mesker

**Affiliations:** 1 Department of Surgery, Leiden University Medical Center, Albinusdreef 2, 2333 ZA Leiden, The Netherlands; 2 Department of Surgery, VieCuri Medical Center, Venlo, The Netherlands; 3 Department of Pathology, Antonius Hospital, Nieuwegein, The Netherlands; 4 Department of Geriatrics and Gerontology, Leiden University Medical Center, Leiden, The Netherlands

**Keywords:** Colon cancer, Disseminated tumor cells, Tumor microenvironment, Tumor-stroma ratio

## Abstract

**Purpose:**

Current TNM staging does not appropriately identify high-risk colorectal cancer (CRC) patients. The aim of this study was to evaluate whether the presence of disseminated tumor cells (DTCs) in the bone marrow (BM) and the presence of stroma in the primary tumor, i.e., the tumor-stroma ratio (TSR), in patients undergoing surgical resection of primary CRC provides information relevant for disease outcome.

**Methods:**

Patients with primary CRC (*n* = 125), consecutively admitted for curative resection between 2001 and 2007, were included in the study. All patients underwent BM aspiration before surgery. Detection of tumor cells was performed using immunocytochemical staining for cytokeratin (CK-ICC). The TSR was determined on diagnostic H&E stained sections of primary tumors.

**Results:**

DTCs were detected in the BM of 23/125 patients (18 %). No association was found between BM status and overall survival (HR 0.97 (95 % CI 0.45–2.09), *p* = 0.93). Also, no significant difference was found in their 5-year survival rate (resp. 72 % and 68 % for BM-positive versus BM-negative patients). The TSR was found to be associated with a worse overall survival (HR 2.16, 95 % CI 1.02–4.57, *p* = 0.04) with 5-year survival rates of 84 % versus 62 % for stroma-low and stroma-high patients, respectively. No relation was found between the presence of DTCs and TSR.

**Conclusions:**

Our data indicate that the presence of DTCs in the BM of CRC patients is not associated with disease outcome. The TSR was, however, found to be associated with a worse overall survival, which indicates that for CRC the tumor microenvironment plays an important role in its behavior and prognosis.

## Introduction

There is a need for predictors of recurrence of disease after resection of colorectal cancer (CRC) with curative intent. If tumor recurrence can be identified early enough, potentially curative reoperations may be considered. Moreover, selective adjuvant treatment may be administered to patients at a high risk of recurrence.

In CRC the prognosis and indication for adjuvant therapy is mainly based on TNM staging. Although this histopathological approach is of paramount importance in cancer classification, for most CRC patients with stage II disease who are classified as standard risk, there are no additional markers to further refine risk assessment or to predict adjuvant chemotherapy benefit. On the other hand, subgroups of stage III CRC patients may not require postoperative adjuvant chemotherapy and may, thus, be prevented from chemotherapy toxicity and side effects [1]. This implies that current staging does not appropriately identify all high-risk patients, especially in the group of lymph node-negative cases (those presumed to have localized disease only), of which 25 % does exhibit recurrence of disease [2].

Available data strongly support the view that disseminated tumor cells (DTCs) may serve as a candidate biomarker suitable for prognostication in (colorectal) cancer [3, 4]. Bone marrow (BM) appears to be a common homing site for carcinomas derived from different organs, and may serve as a reservoir of DTCs with the capacity to metastasize to other distant organs [4–6]. In breast cancer, for example, the presence of DTCs is being considered as an independent prognostic factor for reduced survival [7]. There are several methods to detect DTCs [8], of which immunocytochemical staining for cytokeratin (CK-ICC) is the most frequently used and most standardized technique. Earlier studies have indicated that the clinical importance of the detection of DCTs in the BM of CRC patients is still a matter of debate [3, 9].

Before tumor cells become DTCs, they escape from their microenvironment. Nowadays, there is an increasing appreciation of the importance of the tumor microenvironment, including the stromal compartment, in the process of tumorigenesis. This compartment facilitates the survival and proliferation of neoplastic cells and promotes epithelial-mesenchymal transition (EMT), as well as local and metastatic dissemination. Moreover, the stroma of each tumor is different in terms of quantity and cellular composition. In recent years, the tumor stroma has gained interest in the clinic with regard to patient prognosis and its potential to affect therapy responses. In colon cancer patients the tumor-stroma ratio (TSR) has been identified in several studies as an important prognosticator of disease-free and overall survival [10–13].

The aim of this study was to determine the clinical importance of DTCs in the BM of patients with primary CRC using immunocytochemical staining for cytokeratin (CK-ICC). We also compared the CK-ICC data to the TSR data. In addition, we evaluated possible relationships between the TSR and DTCs.

## Methods

### Patients

A total of 125 consecutive patients were enrolled in the study between May 2001 and November 2007. Only patients with primary CRC (TNM stage I-III) planned for curative resection in the participating hospitals were included. Patients who died within 2 months after surgery were excluded. After surgery all patients underwent routine clinical examination, liver ultrasonography and CEA testing. Forty-two patients with stage III (27/38) or IV (15/18) disease received systemic adjuvant chemotherapy. Follow-up was carried out in all cases and completed until June 2014. Approval of the local Medical Ethical Committees for this study was provided and informed written consent was obtained from all patients.

### Bone marrow aspirations

From all patients 5–10 ml BM was aspirated from both sides of the anterior iliac crest under general anesthesia prior to surgery. Before inserting the needle in the anterior iliac crest, an incision was made into the overlying skin to prevent contamination with skin epithelial cells. Mononuclear cells were isolated from the BM by ficoll gradient centrifugation and aliquoted for the preparation of cytospin-slides for immunocytochemistry.

### Immunocytochemistry and automated microscopy

The cytospin-slides were stained with primary antibodies directed against cytokeratins 8, 18 and 19 (A45-B/B3; Micromet AG, Munich, Germany), or with isotype antibodies directed against an irrelevant antigen as a negative control (MOPC21; BD Pharmingen, Erembodegem, Belgium). A detailed cytokeratin immunocytochemical (CK-ICC) staining protocol has been published before by Pantel et al. [14]. CK-ICC staining results in a red precipitate in the cytoplasm of cytokeratin-positive cells. The slides were counterstained with haematoxylin (Mayer’s Hemalaum; Merck, Darmstadt, Germany) to visualize the nuclei. The slides were analyzed using an ARIOL SL-50 automated microscope® (Applied Imaging Corporation, San Jose, CA). One slide stained for cytokeratin and one negative control slide were analyzed per patient. Detailed features of this approach have been reported before [15].

By combining CK-ICC with automated microscopy, cytokeratin-positive cells were confirmed by an independent pathologist and categorized into tumor cells, candidate tumor cells, apoptotic cells or hematopoietic cells, based on morphological criteria according to the guidelines of the European ISHAGE Working Group for Standardization of Tumour Cell Detection [16]. Candidate tumor cells and apoptotic cells were denoted as cells that did not meet all criteria for a positive tumor cell, but could not unambiguously be defined as being normal. A patient was considered positive if at least one tumor cell, one candidate tumor cell or one apoptotic cell was found.

### Control samples

To validate our technique, we performed CK-ICC staining on BM derived from 20 breast cancer patients and from 29 individuals that were operated because of a benign disease without any evidence of a malignancy until June 2014.

### Determination of the tumor-stroma ratio

Histopathological examination entailed routine microscopic analysis of 5 μm H&E stained sections of the primary tumor as reported before [10]. The slides were selected from the most invasive part of the tumor (i.e., the slides used in routine pathology to determine the T-status), as indicated in the pathology reports, and analyzed by conventional microscopy. In case the pathology information could not be retrieved, all available tumor slides were collected and analyzed [10]. Areas with the largest amount of stroma were selected using a 2.5× or a 5× objective. Areas in which both tumor and stromal tissue were present were selected using a 10× objective, after which the final TSR score was determined. Tumor cells should be present at all borders of the image field(s) to be selected. Two observers (GvP, WM) estimated the TSR in a blinded manner. A third independent pathologist was decisive in case of an inconclusive score or a lack of consensus. Scoring percentages were given *per* tenfold (10 %, 20 %, 30 % etc.) *per* image-field. Since rectal cancer patients are pre-operatively treated with radiotherapy, which influences the intra-tumor stroma, only tissues from colon cancer patients were evaluated for TSR.

### Statistical analyses

Frequencies were described as mean plus standard deviation (SD), or median plus range in case of a non-normal distribution. Patients that were found to have synchronous metastases, defined as metastases found during operation or within 3 months, were excluded from the disease-free survival analyses. The recurrence rate of the patients with a positive CK-ICC score was compared to those with a negative CK-ICC score using Cox regression adjusted for sex and age. Hazard ratios (HRs) were calculated by Cox regression analysis for overall survival and disease-free survival. Overall survival was considered from the day of primary tumor surgery to the day of death or censored at the most recent follow-up date. Disease-free survival was considered from the day of surgery to the day of recurrence, or to the day of death, or censored at the most recent follow-up date. Associations between BM or TSR status and survival were depicted in Kaplan-Meier survival curves. Stroma-high was defined as >50 % stroma and stroma-low as ≤50 % stroma. All analyses were performed using SPSS for Windows (version 23.0, IBM SPSS Inc, Chicago, Ill). *P*-values <0.05 were considered statistically significant.

## Results

The study population comprised 125 patients (65 females and 60 males). No complications of BM aspiration were reported. The patient and tumor characteristics of the study population are listed in Table 1. The median follow-up time from the date of diagnosis of the primary tumor was 6.5 years (range: 0–12 years). Of the 125 patients, 38 developed recurrent disease during follow-up at either single sites (liver (*n* = 20), lung (*n* = 5), peritoneum/lymph node (*n* = 3), bone (*n* = 1)), or at multiple sites (*n* = 9).

**Table 1:** Clinicopathological characteristics of the study population

	All patients	CK-ICC positive	CK-ICC negative	*P* value
	*N* = 125	*N* = 23	*N* = 102	
Gender				
Male	60 (48)	15 (65)	45 (44)	0.055
Female	65 (52)	8 (35)	57 (56)	
Age (years)^a^	69 (41–90)	68 (45–79)	69 (41–90)	0.694
Hospital				
University	67 (54)	17 (74)	50 (49)	0.025*
Affiliated	58 (46)	6 (26)	52 (51)	
Location primary tumor				
Colon	108 (87)	18 (78)	90 (88)	0.175
Rectum	17 (13)	5 (22)	12 (12)	
TNM stage				
I	22 (18)	5 (22)	17 (17)	0.596
II	47 (38)	7 (30)	40 (39)	
III	38 (30)	6 (26)	32 (31)	
IV	18 (14)	5 (22)	13 (13)	
TSR^b^				
Stroma-low	57 (59)	9 (50)	48 (61)	0.282
Stroma-high	40 (41)	9 (50)	31 (39)	
Number of LNs^a^	14 (1–33)	12 (1–20)	14 (1–33)	0.188
Follow-up (years)^a^	6.5 (0–12)	7.0 (0–12)	5.8 (0–12)	0.851
Death	41 (33)	8 (35)	33 (32)	0.501

Data stated in number (%), unless otherwise indicated

^a^Stated in median (range)

^b^Only colon cancer patients evaluated (total *N* = 97)

Abbreviations: *CK-ICC* cytokeratin immunocytochemistry, *TNM* tumor-node-metastasis, *TSR* tumor-stroma ratio, *LNs* lymph nodes

### Prevalence of DTCs in BM

In 23 of the 125 patients (18 %) disseminated tumor cells (DCTs) were found in the BM using CK-ICC. Table 1 shows, next to the clinical parameters of the patients included in the study, the percentage of BM-positive patients per TNM stage. We found that the percentages of BM-positive patients per stage did not differ significantly. Three patients developed bone metastases of which two were classified as BM-positive. The presence of DCTs in the BM was not found to be associated with TSR (*p* = 0.28).

### Control groups

In Tables 2 and 3 the patient characteristics of both control groups are depicted. In 9 of the 20 patients (45 %) operated because of breast cancer, CK-ICC positive cells were detected in the BM, whereas in 2 of the 29 patients (7 %) operated because of benign disease, CK-ICC positive cells were detected in the BM.

**Table 2:** Characteristics of the breast cancer control group

Breast cancer patients	*N* = 20
Age (years), median (range)	56 (40–74)
Stage	
I	14
II	3
III	3
CK-ICC positive	9

Abbreviation: *CK-ICC* cytokeratin immunocytochemistry

**Table 3:** Characteristics of the benign control group

Benign disease	*N* = 28
Sigmoid resection (diverticulitis)	5
Bowel resection (inflammatory bowel disease)	13
Bowel resection (tubulovillous adenoma)	3
Cholecystectomy	4
Benign stenosis duodenum	1
Hernia repair	2
CK-ICC positive	2

Abbreviation: *CK-ICC* cytokeratin immunocytochemistry

### DTCs and survival

**Fig. 1:**
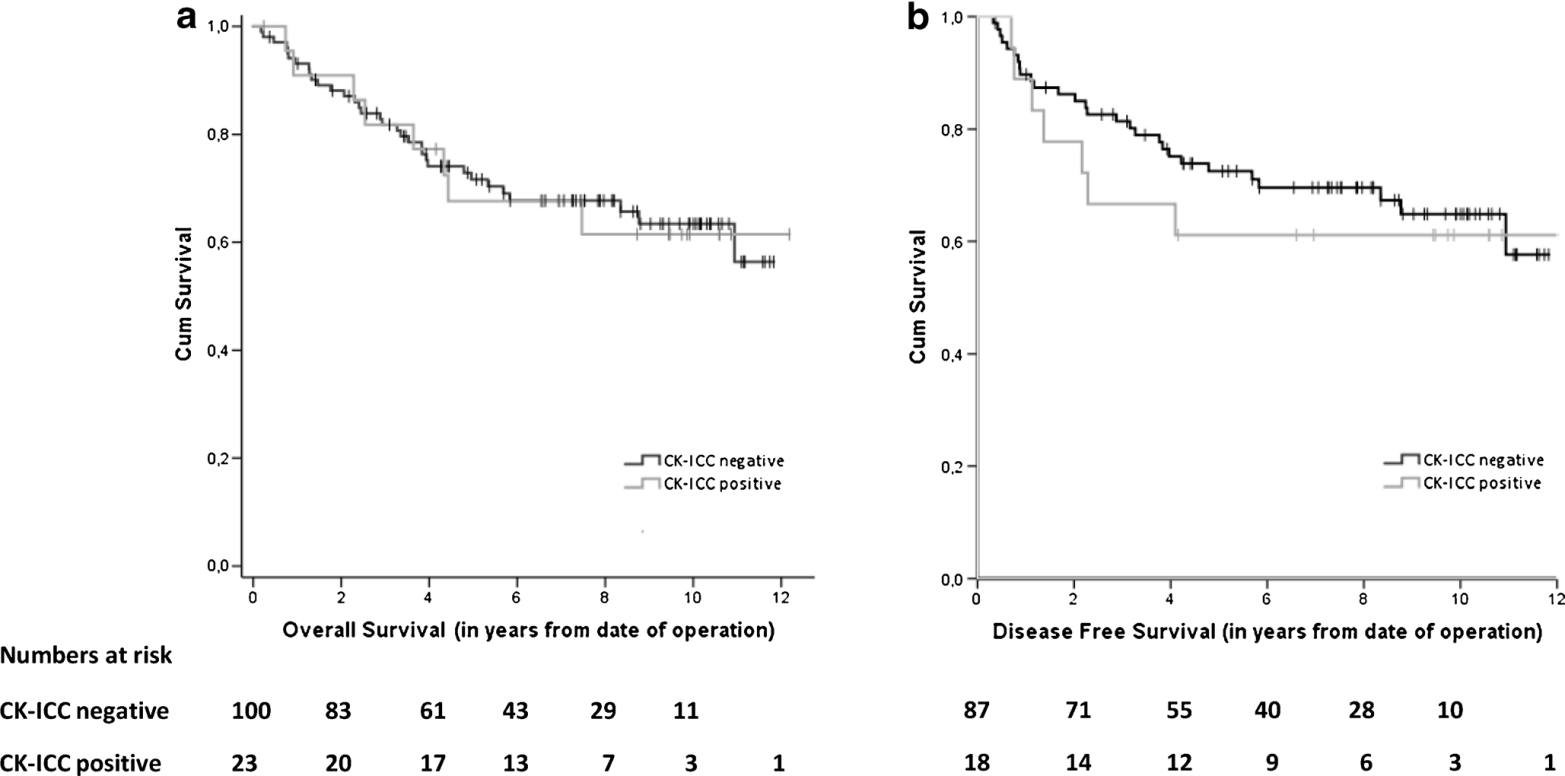
Kaplan-Meier survival curves for CK-ICC negative and CK-ICC positive patients: overall survival (**a**) and disease-free survival (**b**) in patients after surgery for primary colorectal cancer

Patients with a CK-ICC negative BM were not found to exhibit a significantly better overall survival (OS) than those with CK-ICC positive cells in the BM (Fig. 1a); HR 0.97 (95 % CI 0.45–2.09), *p* = 0.93. No significant difference was found in the 5 year survival rate of both groups (68 % and 72 %, respectively). Also, the disease-free survival (DFS) did not show a significant difference between the BM-positive and BM-negative patient groups (Fig. 1b); HR 0.80 (95 % CI 0.35–1.82), *p* = 0.59.

### Subgroup analyses

**Fig. 2:**
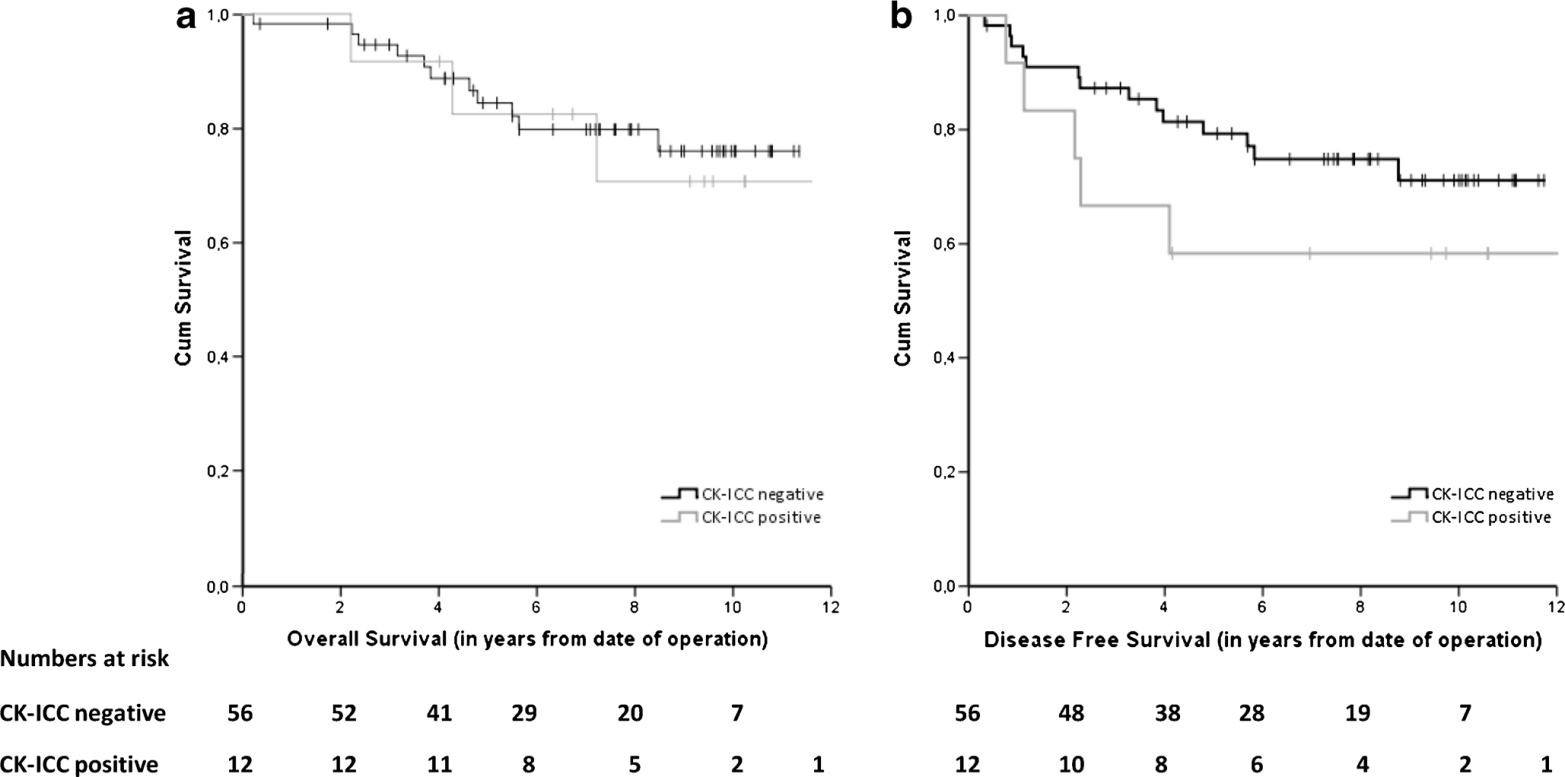
Kaplan-Meier survival curves for CK-ICC negative and CK-ICC positive patients: overall survival (**a**) and disease-free survival (**b**) in lymph node-negative patients after surgery for primary colorectal cancer

In the lymph node-negative (stage I-II) patients, no significant difference was observed between the BM-negative and BM-positive cases in OS or DFS (Fig. 2); HR 0.85 (95 % CI 0.24–3.04), *p* = 0.80 and HR 1.83 (95 % CI 0.66–5.09), *p* = 0.25, respectively. Also in the group of elderly patients (>70 years), no significant difference was found between the BM-negative and BM-positive cases in OS (*n* = 58; *p* = 0.25) or DFS (*n* = 49; *p* = 0.24). Adjustment for sex, age, tumor location and chemotherapy did not change the results for any of these survival analyses.

### TSR and survival

**Fig. 3:**
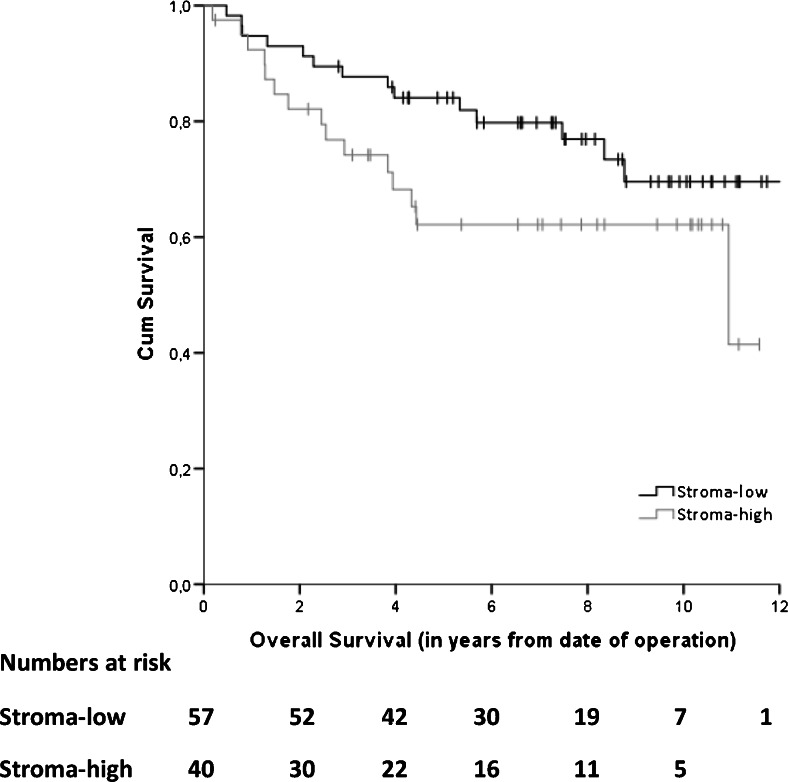
Kaplan-Meier overall survival curve for stroma-low and stroma-high patients after surgery for primary colon cancer

Five out of 108 pre-selected patients could not be analyzed due to a poor quality of the histological material and of 6 patients material was not available, leaving H&E sections from 97 patients for TSR analysis. Fifty-seven of these sections (59 %) were scored as stroma-low and 40 (41 %) as stroma-high. Patients with a high stroma percentage within the primary tumor showed a trend towards a worse OS in an univariate analysis; HR 1.84 (95 % CI 0.89–3.82), *p* = 0.10, with a 5 year survival rate of 84 % versus 62 % in those with a low stroma percentage (Fig. 3). After adjustment for sex, age and chemotherapy, we found that TSR serves as a prognostic factor for a worse OS in patients with a high TSR; HR 2.16 (95 % CI 1.02–4.57), *p* = 0.04. In case of DFS no significant difference between stroma-low and stroma-high patients was observed. The 5 year DFS rates were 76 % and 71 % for the stroma-low and stroma-high cases, respectively.

## Discussion

In nearly one-fifth of the patients with CRC, including early stage cases, we found DTCs in the BM. These DTCs, detected by CK-ICC, were not found to impose a significant impact on the prognosis of patients that underwent resection of the primary tumor. The idea that a high amount of stroma in the primary tumor may be related to the presence of DTCs was not confirmed by our study. We did find, however, that a high stroma-percentage was associated with a worse overall survival in the CRC patients included in this study.

Results obtained by others aimed at assessing the prognostic impact of DTCs in primary CRC cases have so far been conflicting. The amount of positive DTCs in BM were e.g. found to range from 24 to 64 % [6, 17–22]. These differences in (mostly) cytokeratin stained cells may reflect the use of different techniques. Flatmark et al. [23] found that the use of two different techniques (immunomagnetic selection with an anti-EpCAM antibody and CK-ICC) resulted in a minimal detection overlap although, surprisingly, the results of both methods were found to be associated with disease outcome in distinct CRC prognostic subgroups. The main conclusion from their study was that the presence of DTCs in BM may serve as a prognostic biomarker for curatively resected CRC patients.

In some studies, the presence of DTCs was found to be associated with a shorter disease-free survival [18, 24], whereas in other studies no prognostic relevance of DTCs in the BM was noted [6, 19, 21]. Steinert et al. [6] detected the same amount of CK-ICC positive cells throughout all CRC TNM stages, and Lindemann et al. [18] failed to find a significant difference between lymph node-positive and -negative patients, although they did find that the detection of tumor cells in the BM may serve as an independent determinant of relapse. In both studies immunocytochemistry was used for BM analysis. Leinung et al. [17] found an increased percentage of CK-ICC positive cells in the BM in rectal cancer patients compared to colon cancer patients. In 2010, Rahbari et al. published a meta-analysis (in which the above described studies were also included) to assess whether hematogeneous DTCs represent a prognostic factor in patients with CRC. They found a strong prognostic effect of circulating tumor cells in the peripheral blood but, in accordance with our findings, this effect was not found for DCTs in the BM [25].

A pooled analysis of micro-metastases in the BM of breast cancer patients [7] revealed that the presence of DTCs in the BM was predictive for the development of distant metastases after long term follow-up. Tumor cell persistence in BM was also found to be an independent prognostic factor for subsequent breast cancer survival [26]. In contrast to breast cancer, in which bone is one of the most preferential metastatic target sites affecting more than half of the patients during the course of their disease, the incidence of bone metastases in CRC is rare [27, 28]. Three of our 125 patients developed skeletal metastases during follow-up, two of which were BM-positive. In 18 % of the patients DTCs were detected in the BM, but this does not seem to reflect the presence of skeletal metastases. In our control group of 20 breast cancer patients, we found in 45 % of the cases DTCs in the BM. This result is in accordance with other studies reporting DTC occurrences in the BM of 13 to 50 % in early stage breast cancer patients, and increases in patients with metastatic breast cancer of up to 70 % [29]. In 2 of 29 non-carcinoma control cases we found CK-ICC positive DTCs in the BM. This finding is not novel. Depending on the antibody used, CK-positive cells in BM have been found in up to 5.5 % of individuals without a known malignancy [29]. Therefore, it may be questioned whether all disseminated epithelial cells in the BM are truly malignant cells. Although earlier characterization of these cells appeared to be difficult, a malignant phenotype has been suggested [20, 30]. So, the option must be considered that these cells are indeed tumor cells, but that these cells may not all have the capacity to further metastasize. On the other hand, it has recently been found in an experimental model of colon cancer induced by male cancer cells injected into female nude mice that disseminated ‘tumor’ cells (i.e., epithelial-like cells) may be composed of two different populations, one originating from the cancer (cytokeratin-positive, Y chromosome-positive) and one originating from the resident BM cells [31].

The clinical relevance of the molecular detection of DTCs appears to be restricted by both tumor cell heterogeneities [32] and technical limitations. Genomic analyses at a single cell level have shown that DTCs detected with anti-cytokeratin antibodies frequently exhibit heterogeneous tumor-specific aberrations, particularly in patients without overt metastases [33]. Many tumors are known to undergo an extended period of ‘dormancy’, but little is known about the mechanisms underlying the ‘awakening’ of the dormant tumor cells, which may ultimately lead to the formation of metastases. The steady state of dormancy may be disturbed by changes in both DTCs and their surrounding microenvironment [33]. Tumor cells in the BM of CRC patients may be in the “wrong” environment and, as such, be kept in the dormant state [21]. Future efforts towards comprehensive genomic analysis of DTCs may provide a deeper understanding of a clinically relevant biology of DTCs [34].

For many years, tumor stromal formation or desmoplasia was considered to be a passive bystander of tumorigenesis and tumor progression. However, during the last 10 years, attention has shifted towards the tumor microenvironment and it is now well established that tumor stroma plays an important role in cancer initiation and progression. The tumor stroma interacts with nonmalignant cells as well as with malignant cells during different stages of tumorigenesis, ranging from tumor onset to invasion and metastasis [35, 36]. TSR is a relatively simple to apply and cheap cell-based parameter, which is a strong quality. Multiple other studies have confirmed significant prognostic implications of the TSR not only in colon cancer, but also in other solid tumors [37–42]. Further research using a larger prospective cohort should bring this parameter closer to implementation in the TNM classification system.

## Conclusions

DTCs were frequently detected in the BM of CRC patients, but its presence (as based on CK-ICC) did not predict a worse clinical outcome. In the future, a more precise molecular characterization and functional analysis of DTCs in the BM may provide a definite clue to its possible prognostic impact. In the meantime, a pooled analysis of multi-institutional studies on DTCs in the BM of CRC patients may provide more solid information on its prognostic impact. We found that TSR was associated with a worse overall survival in colon cancer patients. Considering its simplicity and availability for conventional clinical pathology, TSR may serve as a new prognostic histological biomarker after its prognostic value has been confirmed in a large prospective study.
